# Supplementary material for: Metastable Oscillatory Modes as a Signature of Entropy Management in the Brain
Source: Entropy (Basel). 2024 Dec 3;26(12):1048. doi: 10.3390/e26121048 (PMC11675728; doi:10.3390/e26121048)
Supplement: Supplementary file 1 [file entropy-26-01048-s001.zip › entropy-3050653-supplementary.pdf]

entropy

MDPI

Supplementary Materials

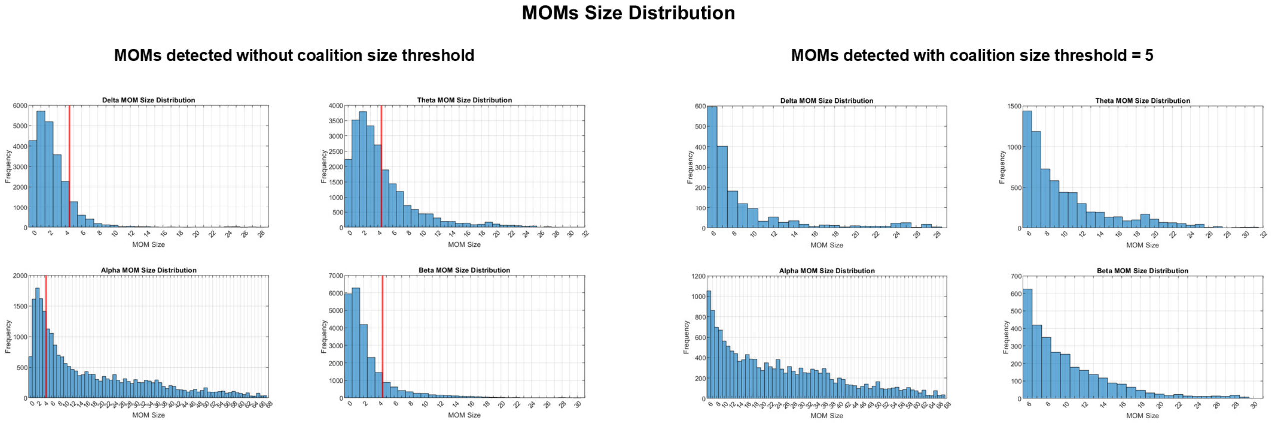

Figure S1. Distribution of MOM coalition sizes across frequency bands (Delta, Theta, Alpha and Beta). The coalition size represents the number of units simultaneously exceeding the power amplitude threshold of 5 standard deviations above baseline. Parameters for this analysis: global coupling strength (K) = 10, mean delay (MD) = 3 ms, and window size = 200 ms. Left panel: total coalition size distribution across frequency bands. The red vertical line indicates the coalition size threshold for MOM detection (>= 5 units). Right panel: distribution of the coalition size of the coalitions engaged in MOMs, defined as exceeding both the power amplitude threshold and the size threshold (>= 5 units).

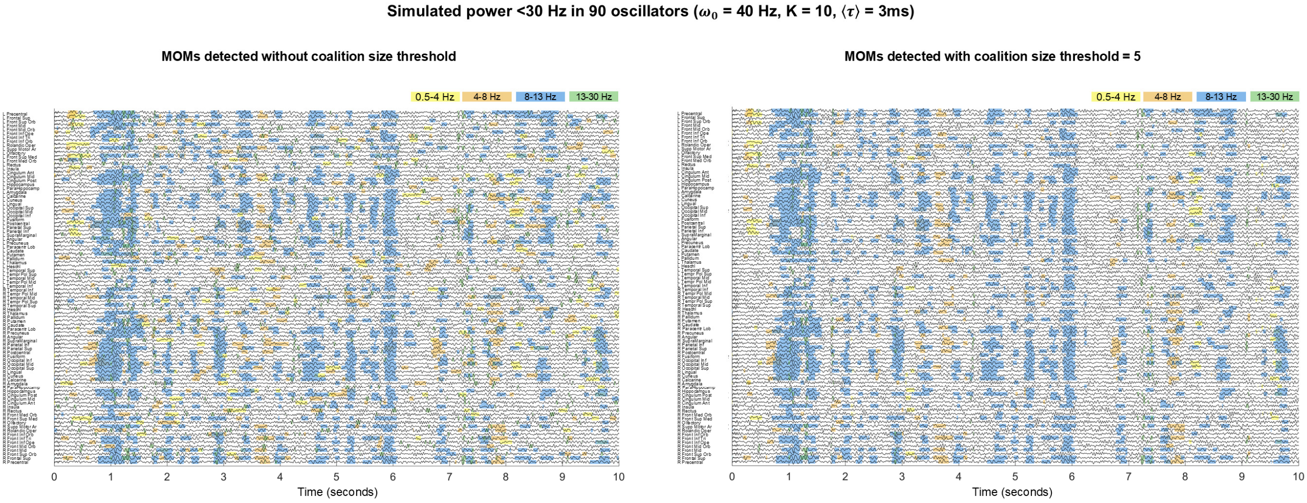

Figure S2. Comparison of MOM detections with and without applying the coalition size threshold. The left panel shows MOMs detected without coalition size filtering, while the right panel shows detections with the coalition size threshold (>= 5 units) applied. The latter demonstrates improved specificity for capturing events involving collective synchronization rather than isolated unit activity. Parameters for this analysis: global coupling strength (K) = 10, mean delay (MD) = 3 ms, window size = 200 ms.

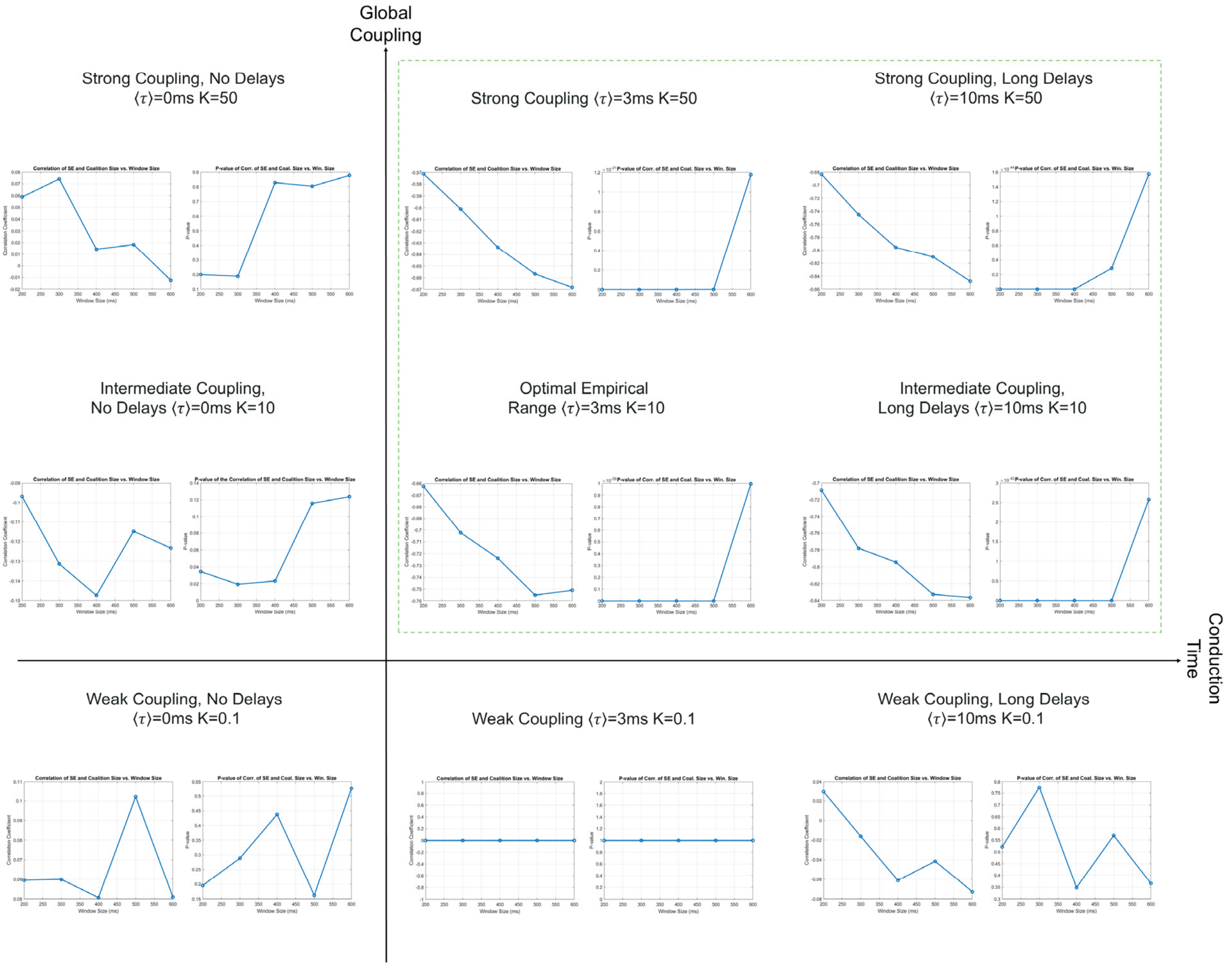

Figure S3. Characterization of the correlation between coalition size and entropy across varying window sizes. Pearson's correlation between the total coalition size across all frequency bands (Delta, Theta, Alpha, Beta) and the Shannon Entropy, calculated with different sliding windows (sizes 200 ms to 600 ms) for different values of global coupling strength (K = 0.1, 10, 50) and mean conduction delay (⟨τ⟩ = 0 ms, 3 ms, 10 ms). Each panel includes two plots: one showing the correlation between Shannon entropy and coalition size vs. window size, and the other showing the corresponding p-value vs. window size. The green dash line highlights the regions of parameters in which correlations are significant (p-value < 0.05, after Bonferroni correction).
